# Supplementary material for: Investigation of Structural Features of Two Related Lipases and the Impact on Fatty Acid Specificity in Vegetable Fats
Source: Int J Mol Sci. 2022 Jun 25;23(13):7072. doi: 10.3390/ijms23137072 (PMC9266812; doi:10.3390/ijms23137072)
Supplement: Supplementary file 1 [file ijms-23-07072-s001.zip › ijms-1771947-supplementary.pdf]

Supplementary figures

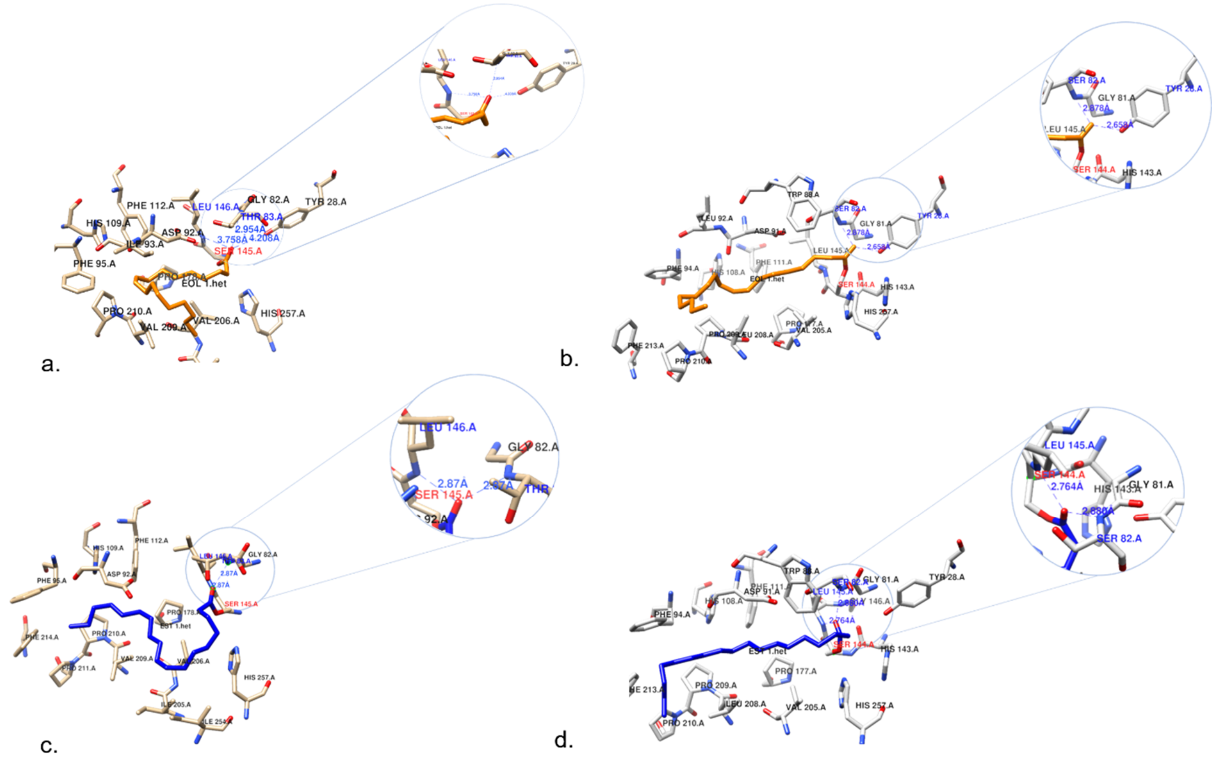

Figure S1. Residues forming hydrogen bond with ligands in the docking simulation. a. ROOL; b. RMOL;c.ROST; d.RMST.

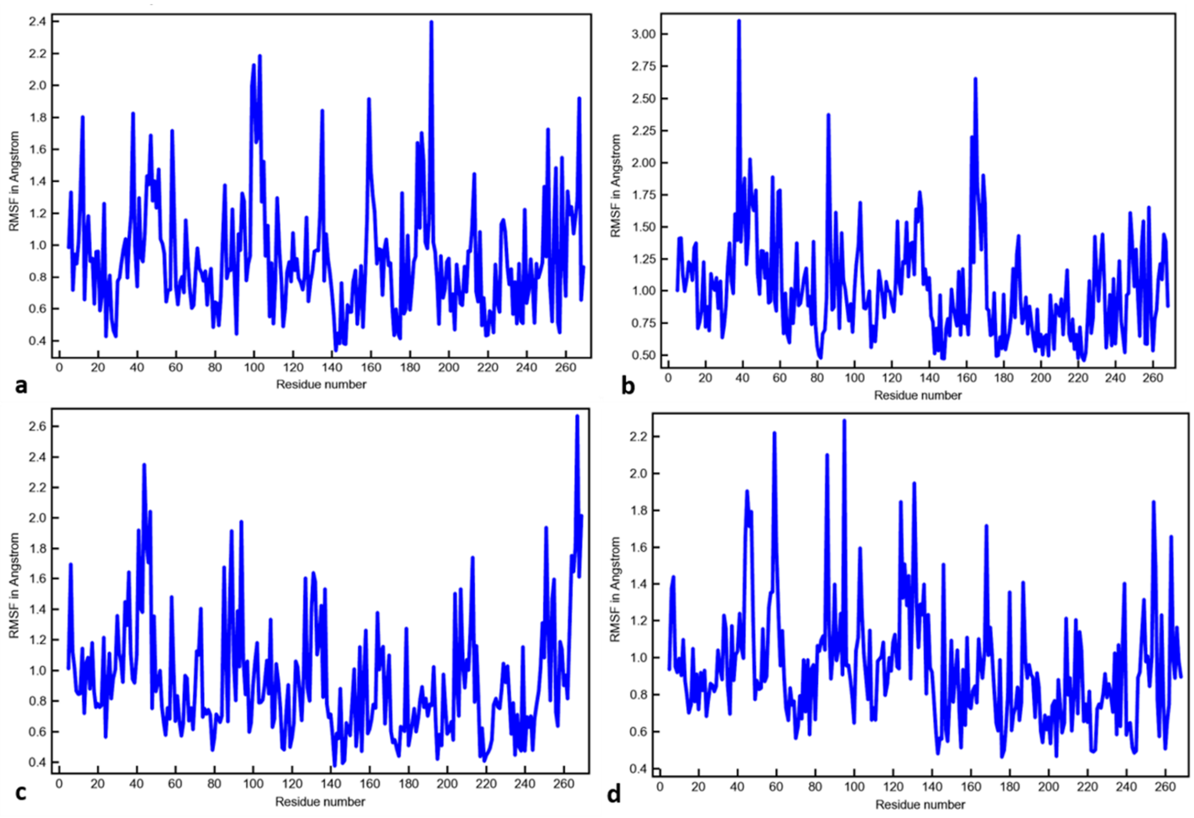

Figure S2. The RMSF per amino acid residue calculated from the average RMSF of the atoms constituting the residue. Plot a. RMOL; b. ROOL; c. RMST; d. ROST.

Supplement file S1: Kinetic and statistic data for transesterification reactions

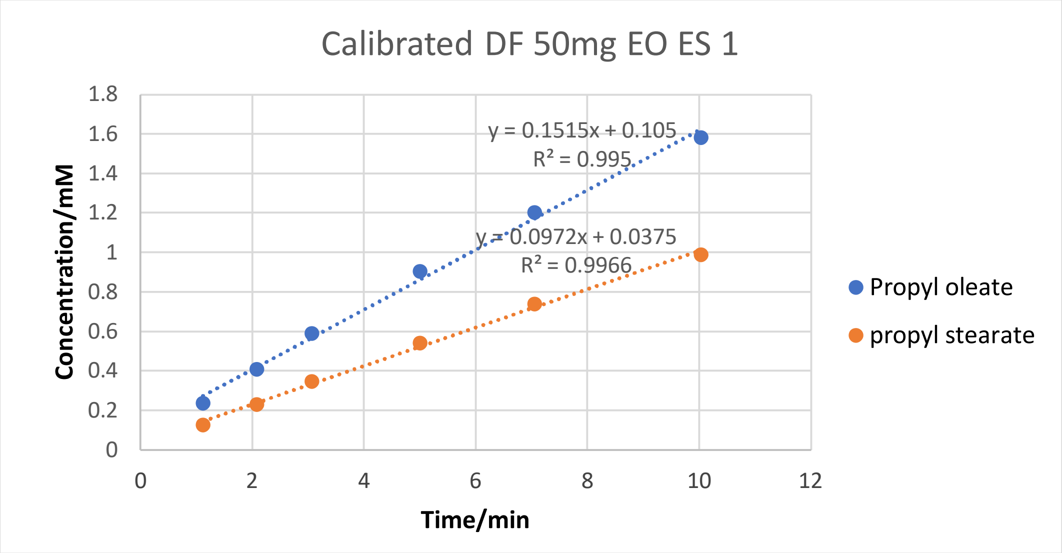

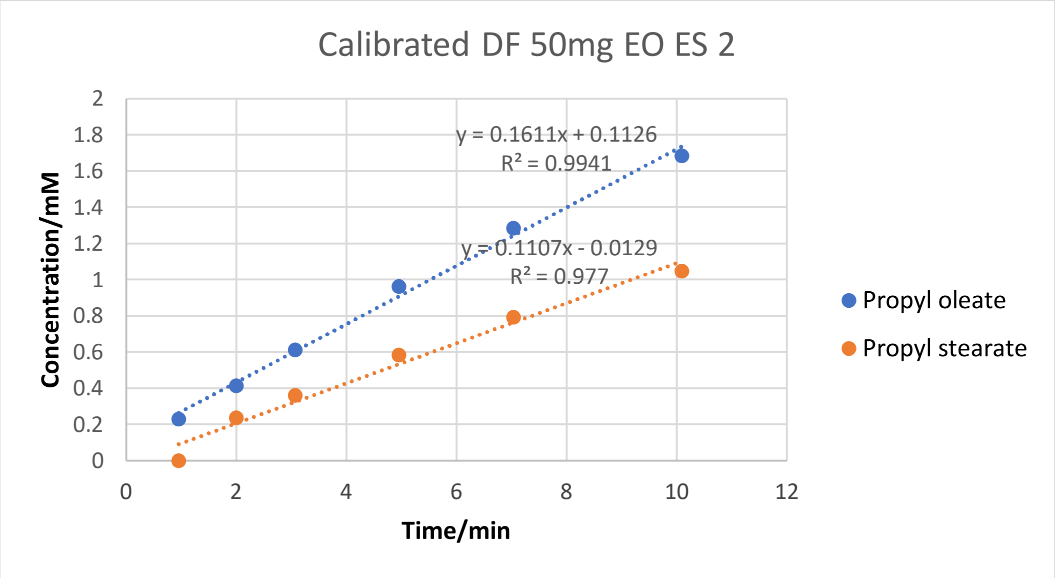

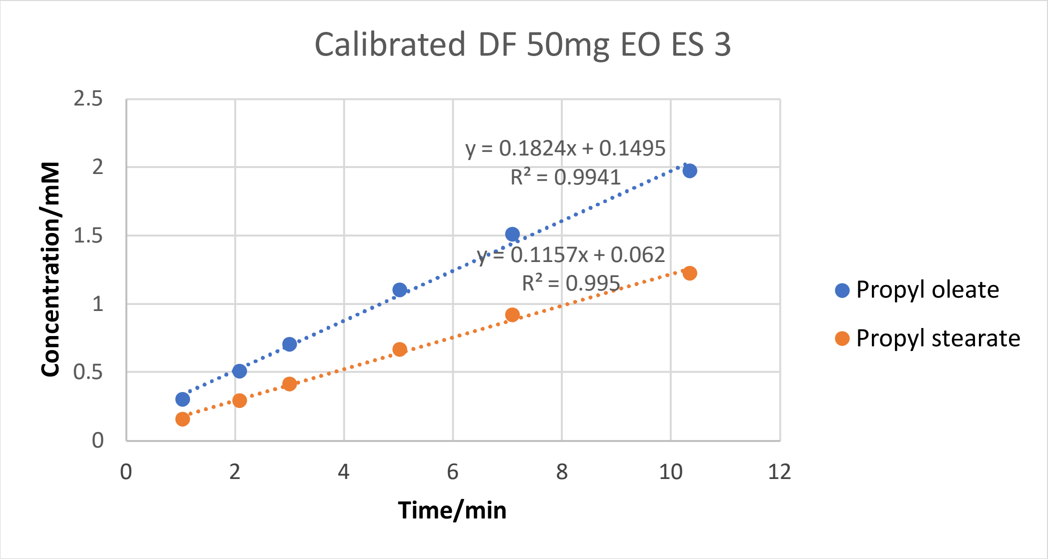

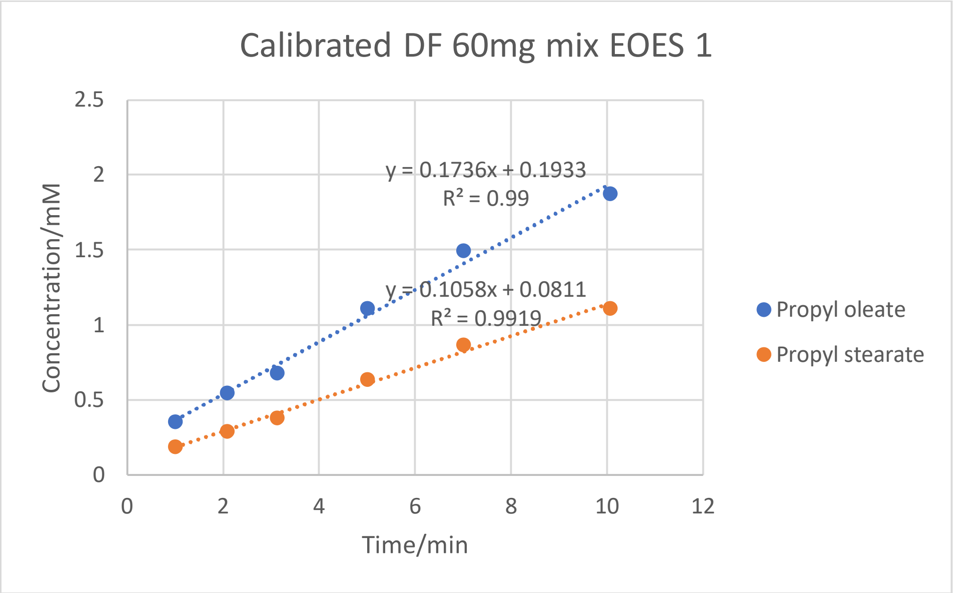
Calibrated DF 60mg mix EOES 1
y = 0.1736x + 0.1933
R² = 0.99
y = 0.1058x + 0.0811
R² = 0.9919
Concentration/mM
Time/min
Propyl oleate
Propyl stearate

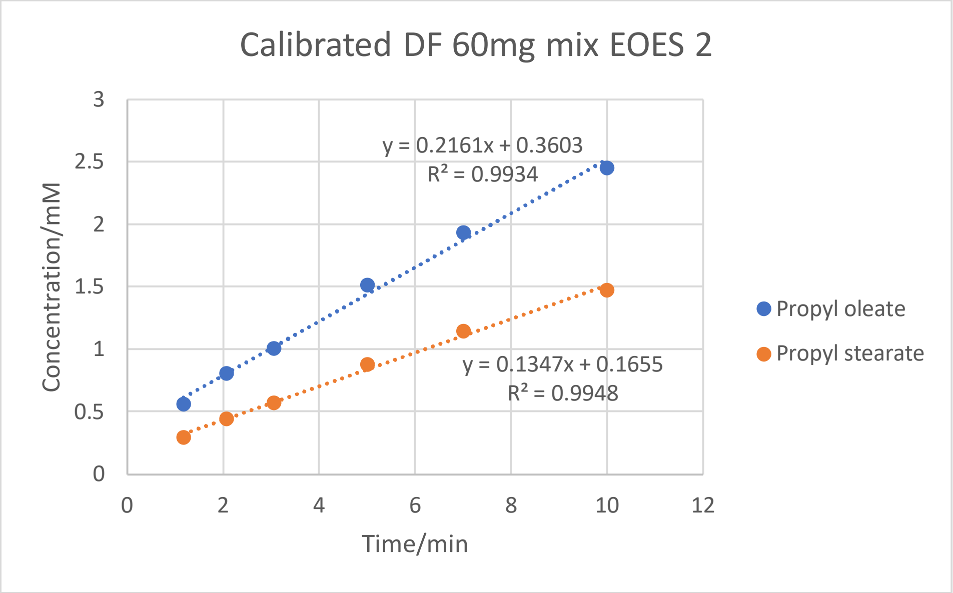
Calibrated DF 60mg mix EOES 2
y = 0.2161x + 0.3603
R² = 0.9934
y = 0.1347x + 0.1655
R² = 0.9948
Concentration/mM
Time/min
Propyl oleate
Propyl stearate

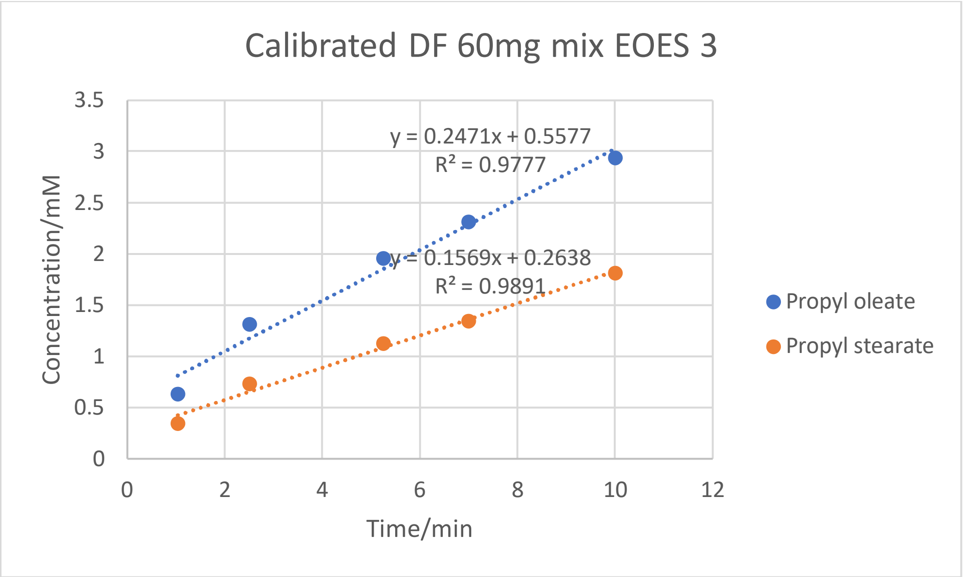

Calibrated DF 60mg mix EOES 3
y = 0.2471x + 0.5577
R² = 0.9777
y = 0.1569x + 0.2638
R² = 0.9891
Concentration/mM
Time/min
0
0.5
1
1.5
2
2.5
3
3.5
0
2
4
6
8
10
12
Propyl oleate
Propyl stearate

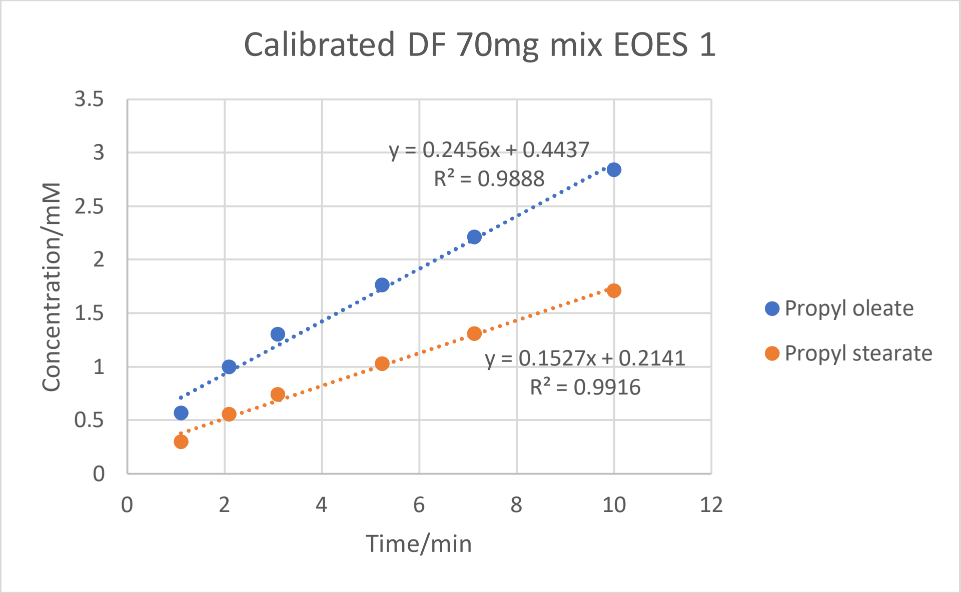
Calibrated DF 70mg mix EOES 1
y = 0.2456x + 0.4437
R² = 0.9888
y = 0.1527x + 0.2141
R² = 0.9916
Concentration/mM
Time/min
Propyl oleate
Propyl stearate

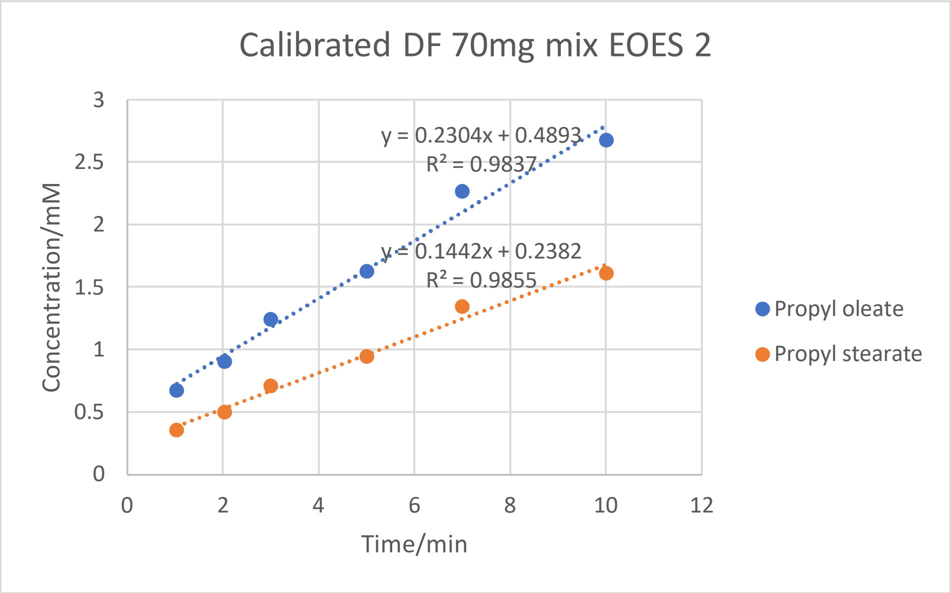
Calibrated DF 70mg mix EOES 2
y = 0.2304x + 0.4893
R² = 0.9837
y = 0.1442x + 0.2382
R² = 0.9855
Concentration/mM
Time/min
Propyl oleate
Propyl stearate

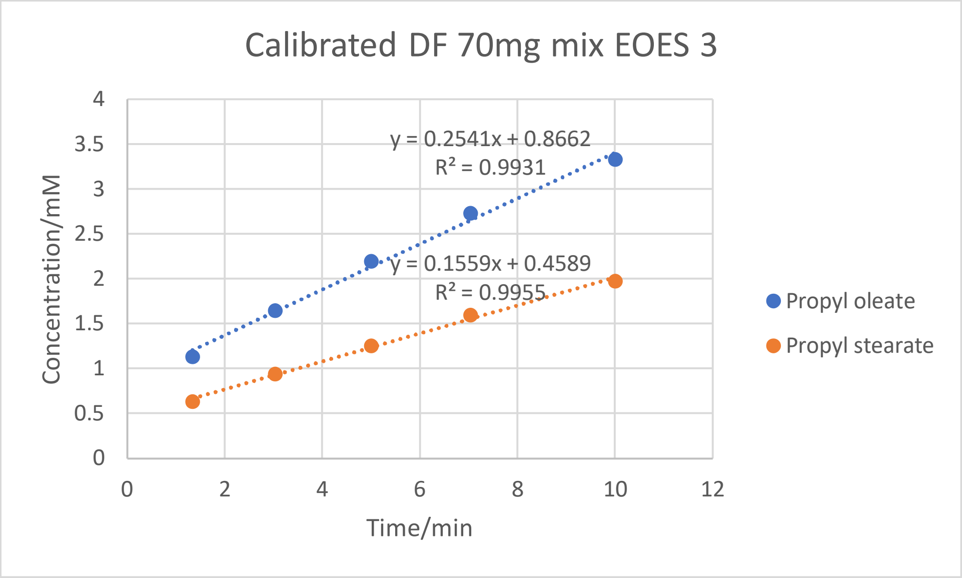
Calibrated DF 70mg mix EOES 3
y = 0.2541x + 0.8662
R² = 0.9931
y = 0.1559x + 0.4589
R² = 0.9955
Concentration/mM
Time/min
Propyl oleate
Propyl stearate

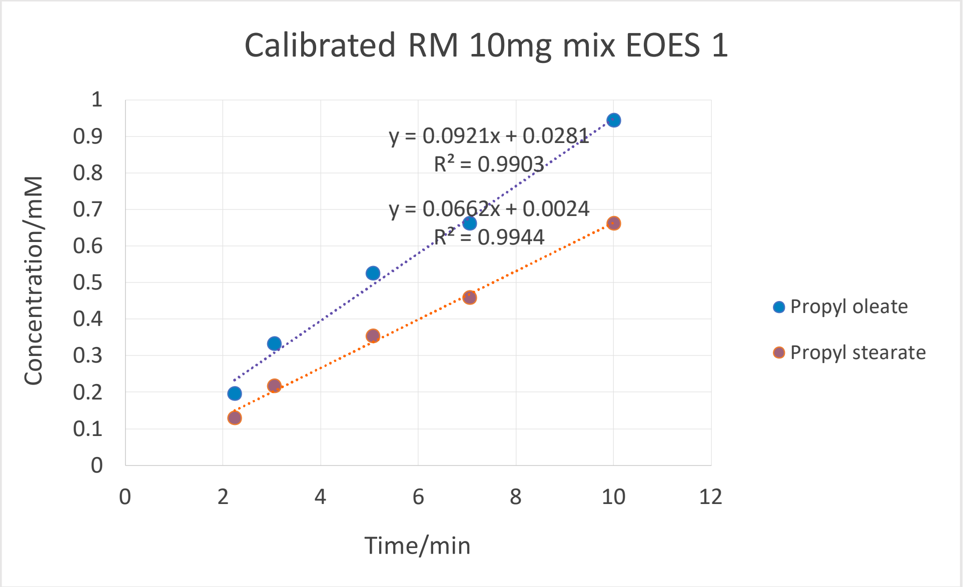
Calibrated RM 10mg mix EOES 1
y = 0.0921x + 0.0281
R² = 0.9903
y = 0.0662x + 0.0024
R² = 0.9944
Concentration/mM
Time/min
Propyl oleate
Propyl stearate

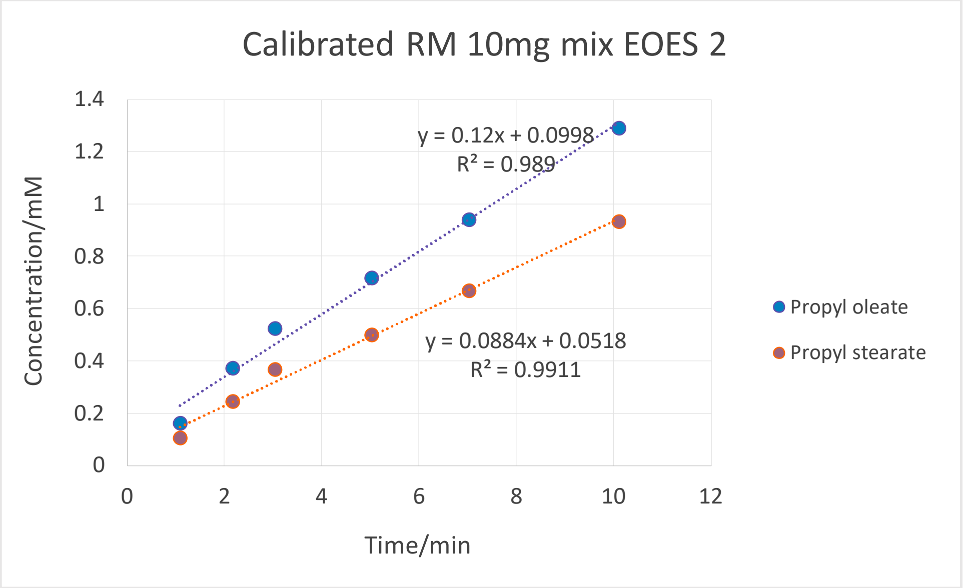
Calibrated RM 10mg mix EOES 2
y = 0.12x + 0.0998
R² = 0.989
y = 0.0884x + 0.0518
R² = 0.9911
Concentration/mM
Time/min
Propyl oleate
Propyl stearate

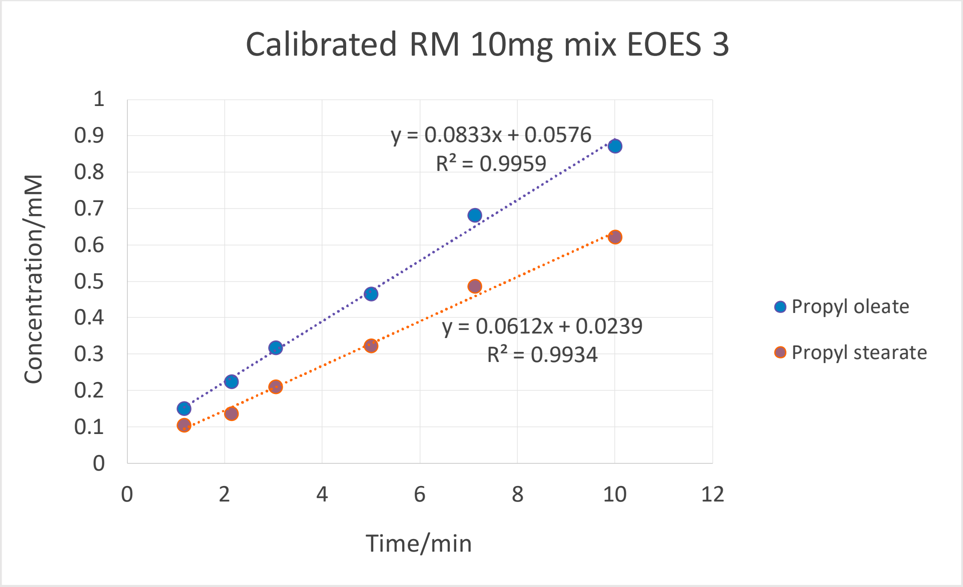
Calibrated RM 10mg mix EOES 3
y = 0.0833x + 0.0576
R² = 0.9959
y = 0.0612x + 0.0239
R² = 0.9934
Concentration/mM
Time/min
Propyl oleate
Propyl stearate

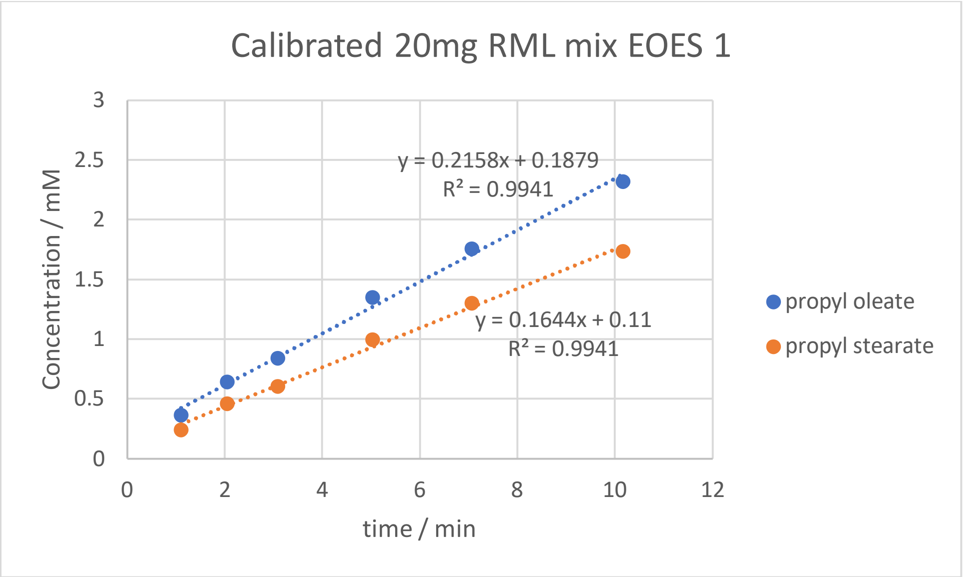
Calibrated 20mg RML mix EOES 1
Concentration / mM
time / min
y = 0.2158x + 0.1879
R² = 0.9941
y = 0.1644x + 0.11
R² = 0.9941
propyl oleate
propyl stearate
0
0.5
1
1.5
2
2.5
3
0
2
4
6
8
10
12

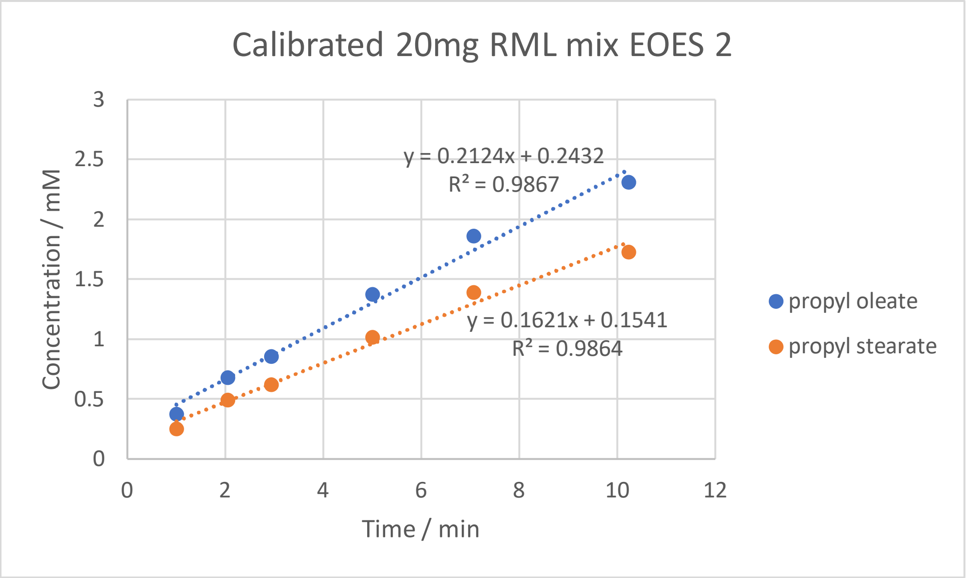
Calibrated 20mg RML mix EOES 2
Concentration / mM
Time / min
y = 0.2124x + 0.2432
R² = 0.9867
y = 0.1621x + 0.1541
R² = 0.9864
propyl oleate
propyl stearate
0
0.5
1
1.5
2
2.5
3
0
2
4
6
8
10
12

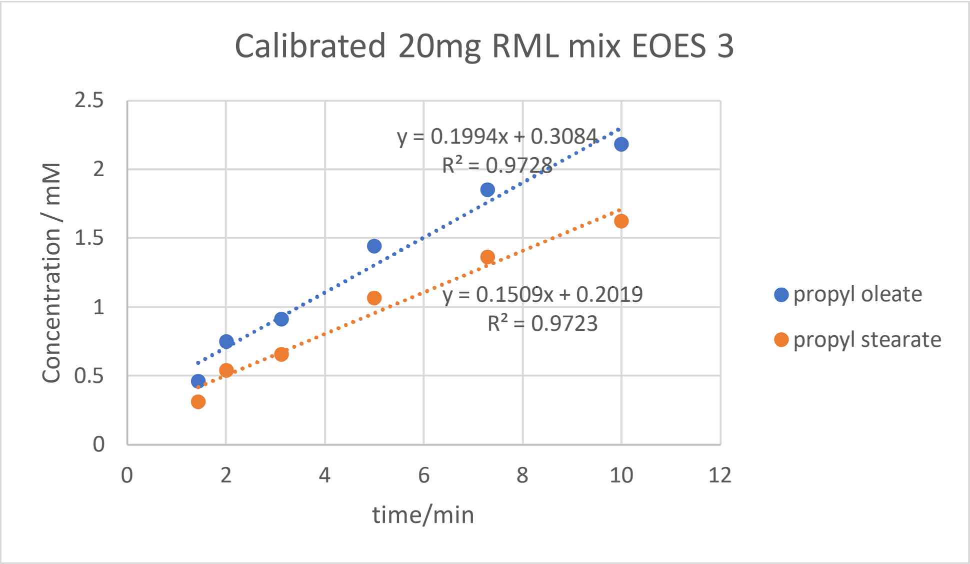
Calibrated 20mg RML mix EOES 3
Concentration / mM
time/min
y = 0.1994x + 0.3084
R² = 0.9728
y = 0.1509x + 0.2019
R² = 0.9723
propyl oleate
propyl stearate
0
0.5
1
1.5
2
2.5
0
2
4
6
8
10
12

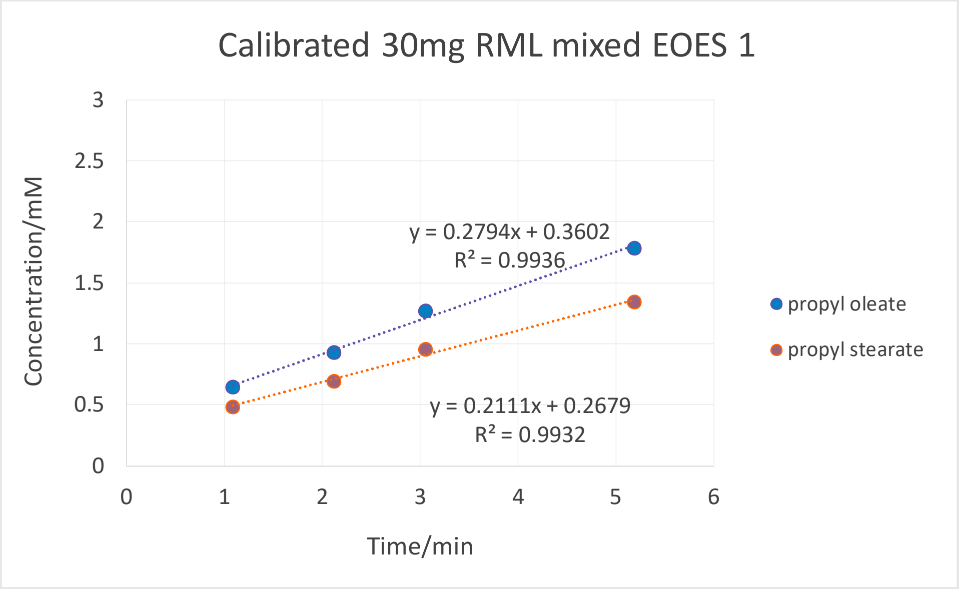

Calibrated 30mg RML mixed EOES 1
Concentration/mM
Time/min
y = 0.2794x + 0.3602
R² = 0.9936
y = 0.2111x + 0.2679
R² = 0.9932
propyl oleate
propyl stearate
0
0.5
1
1.5
2
2.5
3
0
1
2
3
4
5
6

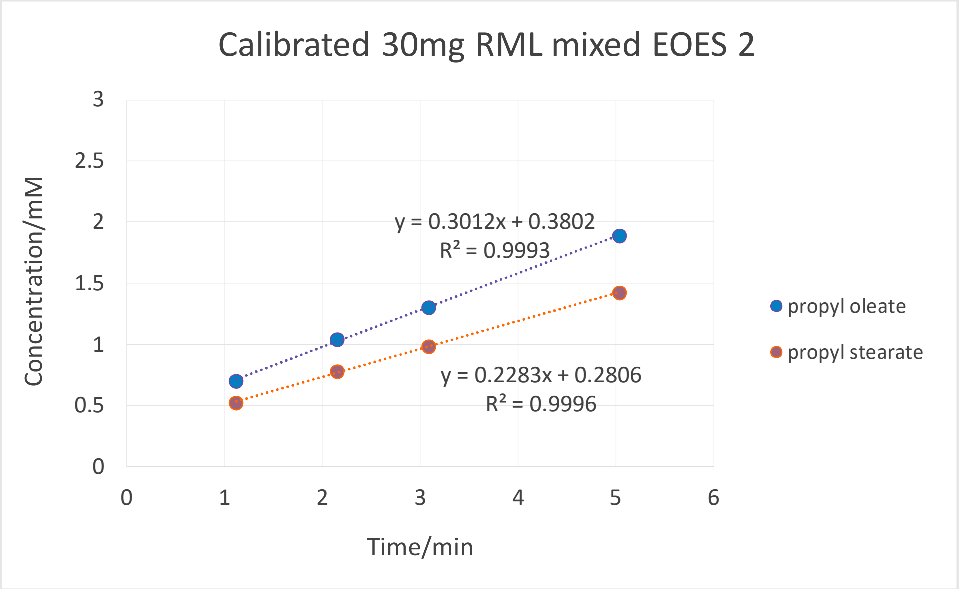

Calibrated 30mg RML mixed EOES 2
Concentration/mM
Time/min
y = 0.3012x + 0.3802
R² = 0.9993
y = 0.2283x + 0.2806
R² = 0.9996
propyl oleate
propyl stearate
0
0.5
1
1.5
2
2.5
3
0
1
2
3
4
5
6

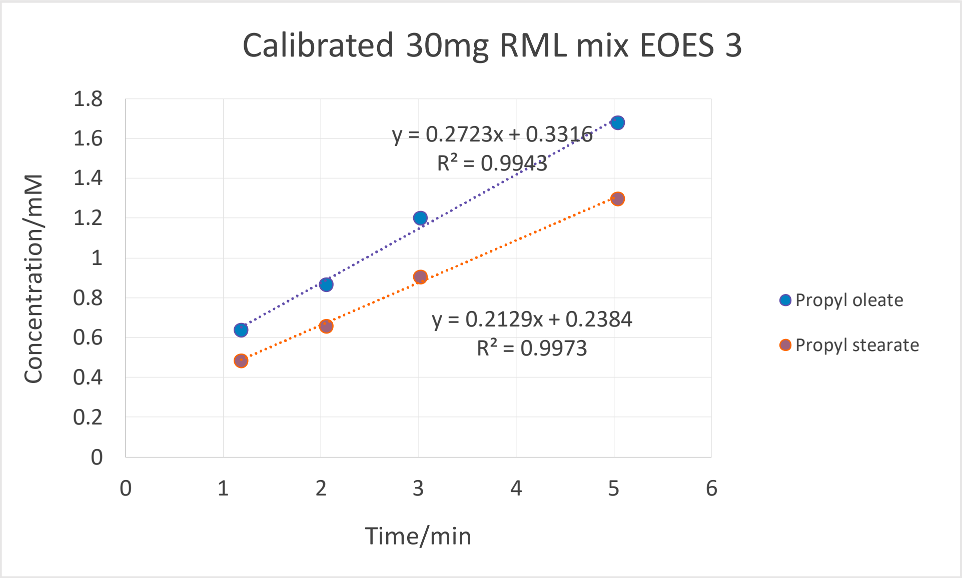

Calibrated 30mg RML mix EOES 3
Concentration/mM
Time/min
y = 0.2723x + 0.3316
R² = 0.9943
y = 0.2129x + 0.2384
R² = 0.9973
Propyl oleate
Propyl stearate
0
0.2
0.4
0.6
0.8
1
1.2
1.4
1.6
1.8
0
1
2
3
4
5
6

Method

μ_1: population mean of ROL(DF)
μ_2: population mean of RML(RM)
Difference: $\mu_1 - \mu_2$

Equal variances are not assumed for this analysis.

Descriptive Statistics

Sample	N	Mean	StDev	SE Mean
ROL(DF)	9	0.6300	0.0255	0.0085
RML(RM)	9	0.7522	0.0186	0.0062

Test

Null hypothesis	H_0: $\mu_1 - \mu_2 = 0$
Alternative hypothesis	H_1: $\mu_1 - \mu_2 \neq 0$

T-Value	DF	P-Value
-12.42	19	0.000

Estimation for Difference

Difference	95% CI for Difference
-0.1222	(-0.1448, -0.0997)

Test

Null hypothesis	H_0: $\mu_1 - \mu_2 = 0$
Alternative hypothesis	H_1: $\mu_1 - \mu_2 \neq 0$

T-Value	DF	P-Value
-11.63	14	0.000

Supplementary file S2

Results from MAUDI Orbitrap MS analysis, DF15 and RM

```
DF:
>1TIC_1|Chains A, B|LIPASE|Rhizopus oryzae (64495)
SDGGKVVAATTAQIQEFTKYAGIAATAYCRSVVPGNKWDCVQCQKWVPDGKIITTFTSLLSD
TNGYVLRSDKQKTIYLVFRGTNSFRSAITDIVFNFSDYKPVKGAKVHAGFLSSYEQVVNDYF
PVVQEQLTAHPTYKVIVTGHSLGGAQALLAGMDLYQREPRLSPKNLSIFTVGGPRVGNPTFA
YYVESTGIPFQRTVHKRDIVPHVPPQSFGFLHPGVESWIKSGTSNVQICTSEIETKDCSNSI
VPFTSILDHLSYFDINEGSCL

RM
>3TGL_1|Chain A|TRIACYL-GLYCEROL ACYLHYDROLASE|Rhizomucor
miehei (4839)
SIDGGIRAATSQEINELTYYTTLSANSYCRTVIPGATWDCIHCDATEDLKIIKTWSTLIYDT
NAMVARGDSEKTIYIVFRGSSSIRNWIADLTFVPVSYPPVSGTKVHKGFLDSYGEVQNELVA
TVLDQFKQYPSYKVAVTGHSLGGATVLLCALDLYQREEGLSSSNLFLYTQGQPRVGDPAFAN
YVVSTGIPYRRTVNERDIVPHLPPAAFGFLHAGEEYWITDNSPETVQVCTSDLETSDCSNSI
VPFTSVLDHLSYFGINTGLCT
```

MALDI MS spectra

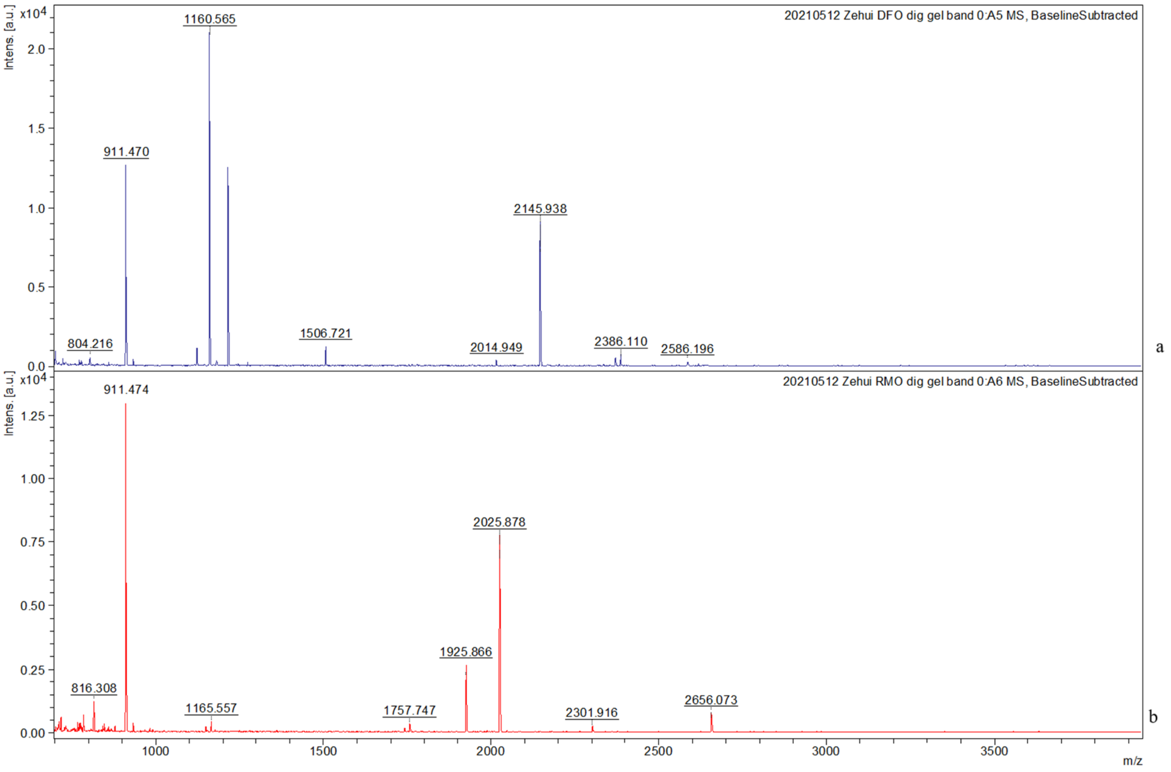

Figure.1 MALDI MS spectra. a. Spectra of sample DF15. b. Spectra of sample RM.

RM sample (ALL peptides with an individual ion score lower than 18 is filtered away):

MASCOT Search Results

Protein View: zehui-lipaseRML

zehui-lipaseRML

**Database:**	Temporary_Database_Proteins
**Score:**	1315
**Nominal mass (M_r):**	39577
**Calculated pI:**	4.92

Sequence similarity is available as an NCBI BLAST search of zehui-lipaseRML against nr.

Search parameters

**MS data file:**	\\MASCOT23AN\Users\User folders\Katja\Projects\Project Zehui\Orbi 210517\210512_Zehui_RM_MassRange_300_1300.raw
**Enzyme:**	Trypsin: cuts C-term side of KR unless next residue is P.
**Variable modifications:**	Carbamidomethyl (C), Oxidation (M)

Protein sequence coverage: 42%

Matched peptides shown in ***bold red***.

```
  1  MVLKQRANYL  GFLIVFFTAF  LVEAVPIKRQ  SNSTVDSLPP  LIPSRTSAPS
 51  SSPSTTDPEA  PAMSRNGPLP  SDVETKYGMA  LNATSYPDSV  VQAMSIDGGI
```
101 R**AATSQEINE** **LTYYTTLSAN** **SYCRTVIPGA** **TWDCIHCDAT** **EDLK**IIK**TWS**
151 **TLIYDTNAMV** **ARGDSEKTIY** **IVFR**GSSSIR **NWIADLTFVP** **VSYPPVSGTK**
201 VHKGFLDSYG EVQNELVATV LDQFK**QYPSY** **KVAVTGHSLG** **GATALLCALD**
251 **LYQREEGLSS** **SNLFLYTQGQ** **PRVGDPAFAN** **YVVSTGIPYR** **R**TVNERDIVP
301 HLPPAAFGFL HAGEEYWITD NSPETVQVCT SDLETSDCSN SIVPFTSVLD
351 HLSYFGINTG LCT

Query	Start	–	End	Observed	Mr(expt)	Mr(calc)	ppm	M	Score	Expect	Rank	U	Peptide
4184	102	–	124	886.0771	2655.2094	2655.2122	-1.06	0	55	3.4e-006	1	U	R.AATSQEINELTYYTTLSANSYCR.T + Carbamidomethyl (C)
4185	102	–	124	886.0778	2655.2115	2655.2122	-0.27	0	40	9.5e-005	1	U	R.AATSQEINELTYYTTLSANSYCR.T + Carbamidomethyl (C)
3644	125	–	144	768.0219	2301.0439	2301.0406	1.44	0	22	0.0069	1	U	R.TVIPGATWDCIHCDATEDLK.I + 2 Carbamidomethyl (C)
2028	148	–	162	871.4320	1740.8494	1740.8505	-0.65	0	63	4.7e-007	1	U	K.TWSTLIYDTNAMVAR.G
2029	148	–	162	871.4321	1740.8496	1740.8505	-0.49	0	54	3.8e-006	1	U	K.TWSTLIYDTNAMVAR.G
2033	148	–	162	581.2909	1740.8509	1740.8505	0.23	0	46	2.5e-005	1	U	K.TWSTLIYDTNAMVAR.G

Query	Start	–	End	Observed	Mr(expt)	Mr(calc)	ppm	M	Score	Expect	Rank	U	Peptide
2035	148	–	162	581.2911	1740.8515	1740.8505	0.60	0	47	1.8e-005	1	U	K.TWSTLIYDTNAMVAR.G
2110	148	–	162	879.4293	1756.8440	1756.8454	-0.80	0	76	2.5e-008	1	U	K.TWSTLIYDTNAMVAR.G + Oxidation (M)
2126	148	–	162	879.4299	1756.8452	1756.8454	-0.11	0	62	5.8e-007	1	U	K.TWSTLIYDTNAMVAR.G + Oxidation (M)
2139	148	–	162	586.6231	1756.8474	1756.8454	1.12	0	41	7.1e-005	1	U	K.TWSTLIYDTNAMVAR.G + Oxidation (M)
2141	148	–	162	586.6231	1756.8475	1756.8454	1.21	0	40	0.00011	1	U	K.TWSTLIYDTNAMVAR.G + Oxidation (M)
1282	163	–	174	476.5891	1426.7456	1426.7456	-0.041	1	22	0.0057	1	U	R.GDSEKTIYIVFR.G
1283	163	–	174	476.5893	1426.7461	1426.7456	0.34	1	22	0.0059	1	U	R.GDSEKTIYIVFR.G
336	168	–	174	456.2708	910.5271	910.5276	-0.58	0	44	4.4e-005	1	U	K.TIYIVFR.G
341	168	–	174	456.2711	910.5277	910.5276	0.059	0	37	0.00021	1	U	K.TIYIVFR.G
342	168	–	174	456.2711	910.5277	910.5276	0.13	0	18	0.015	1	U	K.TIYIVFR.G
3325	181	–	200	1096.0744	2190.1342	2190.1361	-0.86	0	52	5.8e-006	1	U	R.NWIADLTFVPVSYPPVSGTK.V
3329	181	–	200	731.0526	2190.1359	2190.1361	-0.083	0	46	2.3e-005	1	U	R.NWIADLTFVPVSYPPVSGTK.V
3330	181	–	200	731.0526	2190.1361	2190.1361	-0.00091	0	52	6.3e-006	1	U	R.NWIADLTFVPVSYPPVSGTK.V
3331	181	–	200	1096.0771	2190.1396	2190.1361	1.61	0	60	1e-006	1	U	R.NWIADLTFVPVSYPPVSGTK.V
124	226	–	231	393.1962	784.3778	784.3755	2.90	0	20	0.011	1	U	K.QYPSYK.V
125	226	–	231	393.1970	784.3794	784.3755	4.96	0	27	0.002	1	U	K.QYPSYK.V
3879	232	–	254	796.0897	2385.2472	2385.2475	-0.11	0	45	3.2e-005	1	U	K.VAVTGHSLGGATALLCALDLYQR.E + Carbamidomethyl (C)
3880	232	–	254	796.0900	2385.2481	2385.2475	0.25	0	55	3e-006	1	U	K.VAVTGHSLGGATALLCALDLYQR.E + Carbamidomethyl (C)
2937	255	–	272	675.9983	2024.9731	2024.9803	-3.55	0	25	0.0034	1	U	R.EEGLSSSNLFLYTQGQPR.V
2946	255	–	272	1013.4962	2024.9778	2024.9803	-1.22	0	92	7e-010	1	U	R.EEGLSSSNLFLYTQGQPR.V
2954	255	–	272	1013.4964	2024.9782	2024.9803	-1.02	0	92	7e-010	1	U	R.EEGLSSSNLFLYTQGQPR.V
2955	255	–	272	1013.4965	2024.9784	2024.9803	-0.92	0	24	0.004	1	U	R.EEGLSSSNLFLYTQGQPR.V
2960	255	–	272	676.0003	2024.9790	2024.9803	-0.63	0	30	0.00097	1	U	R.EEGLSSSNLFLYTQGQPR.V
2974	255	–	272	1013.4971	2024.9796	2024.9803	-0.33	0	43	5e-005	1	U	R.EEGLSSSNLFLYTQGQPR.V
2983	255	–	272	676.0008	2024.9805	2024.9803	0.082	0	21	0.0078	1	U	R.EEGLSSSNLFLYTQGQPR.V
2704	273	–	290	963.4880	1924.9614	1924.9683	-3.58	0	89	1.2e-009	1	U	R.VGDPAFANYVVSTGIPYR.R
2707	273	–	290	963.4903	1924.9661	1924.9683	-1.14	0	78	1.5e-008	1	U	R.VGDPAFANYVVSTGIPYR.R
2708	273	–	290	642.6627	1924.9663	1924.9683	-1.05	0	37	0.00021	1	U	R.VGDPAFANYVVSTGIPYR.R
2714	273	–	290	642.6630	1924.9671	1924.9683	-0.66	0	30	0.0011	1	U	R.VGDPAFANYVVSTGIPYR.R
2721	273	–	290	963.4911	1924.9677	1924.9683	-0.34	0	62	6.4e-007	1	U	R.VGDPAFANYVVSTGIPYR.R
2727	273	–	290	963.4914	1924.9682	1924.9683	-0.070	0	48	1.7e-005	1	U	R.VGDPAFANYVVSTGIPYR.R
3118	273	–	291	521.2743	2081.0679	2081.0694	-0.72	1	46	2.5e-005	1	U	R.VGDPAFANYVVSTGIPYRR.T
3129	273	–	291	521.2745	2081.0691	2081.0694	-0.18	1	46	2.7e-005	1	U	R.VGDPAFANYVVSTGIPYRR.T

DF sample (ALL peptides with an individual ion score lower than 18 is filtered away):

MASCOT Search Results

Protein View: Zehui_Lipadyou_2_200813

Zehui_Lipadyou_2_200813

**Database:**	Temporary_Database_Proteins
**Score:**	3026
**Nominal mass (M_r):**	42112
**Calculated pI:**	7.06

Sequence similarity is available as an NCBI BLAST search of Zehui_Lipadyou_2_200813 against nr.

Search parameters

**MS data file:**	\\MASCOT23AN\Users\User folders\Katja\Projects\Project Zehui\Orbi 210517\210512_Zehui_DF_MassRange_300_1300.raw
**Enzyme:**	Trypsin: cuts C-term side of KR unless next residue is P.
**Variable modifications:**	Carbamidomethyl (C), Oxidation (M)

Protein sequence coverage: 68%

Matched peptides shown in ***bold red***.

```
  1 MVSFISISQG VSLCLLVSSM MLGSSAVPVS GKSGSSNTAV SASDNAALPP
 51 LISSRCAPPS NKGSKSDLQA EPYNMQKNTE WYESHGGNLT SIGKRDDNLV
101 GGMTLDLPSD APPISLSSST NSASDGGKVV AATTAQIQEF TKYAGIAATA
151 YCRSVVPGNK WDCVQCQKWV PDGKIITTFT SLLSDTNGYV LRSDKQKTIY
201 LVFRGTNSFR SAITDIVFNF SDYKPVKGAK VHAGFLSSYE QVVNDYFPVV
251 QEQLTAHPTY KVIVTGHSLG GAQALLAGMD LYQREPRLSP KNLSIFTVGG
301 PRVGNPTFAY YVESTGIPFQ RTVHKRDIVP HVPPQSFGFL HPGVESWIKS
351 GTSNVQICTS EIETKDCSNS IVPFTSILDH LSYFDINEGS CL
```

Query	Start	–	End	Observed	Mr(expt)	Mr(calc)	ppm	M	Score	Expect	Rank	U	Peptide
3506	33	–	55	734.7050	2201.0932	2201.0924	0.39	0	19	0.012	1	U	K.SGSSNTAVSASDNAALPPLISSR.C
4994	96	–	128	1073.5051	3217.4935	3217.4933	0.067	0	37	0.00019	1	U	R.DDNLVGGMTLDLPSDAPPISLSSSTNSASDGGK.V
4995	96	–	128	1073.5056	3217.4950	3217.4933	0.53	0	59	1.2e-006	1	U	R.DDNLVGGMTLDLPSDAPPISLSSSTNSASDGGK.V
5007	96	–	128	809.3758	3233.4741	3233.4882	-4.35	0	31	0.00074	1	U	R.DDNLVGGMTLDLPSDAPPISLSSSTNSASDGGK.V + Oxidation (M)
5012	96	–	128	1078.8344	3233.4814	3233.4882	-2.10	0	29	0.0014	1	U	R.DDNLVGGMTLDLPSDAPPISLSSSTNSASDGGK.V + Oxidation (M)
5018	96	–	128	1078.8355	3233.4847	3233.4882	-1.08	0	53	5.4e-006	1	U	R.DDNLVGGMTLDLPSDAPPISLSSSTNSASDGGK.V + Oxidation (M)
5019	96	–	128	1078.8356	3233.4850	3233.4882	-0.99	0	45	3.2e-005	1	U	R.DDNLVGGMTLDLPSDAPPISLSSSTNSASDGGK.V + Oxidation (M)
1549	129	–	142	753.9099	1505.8052	1505.8090	-2.48	0	58	1.6e-006	1	U	K.VVAATTAQIQEFTK.Y
1550	129	–	142	502.9432	1505.8079	1505.8090	-0.71	0	37	0.00019	1	U	K.VVAATTAQIQEFTK.Y
1551	129	–	142	753.9113	1505.8080	1505.8090	-0.65	0	79	1.3e-008	1	U	K.VVAATTAQIQEFTK.Y
1552	129	–	142	753.9113	1505.8081	1505.8090	-0.54	0	30	0.0011	1	U	K.VVAATTAQIQEFTK.Y
1556	129	–	142	753.9114	1505.8083	1505.8090	-0.43	0	23	0.0054	1	U	K.VVAATTAQIQEFTK.Y
1557	129	–	142	753.9114	1505.8083	1505.8090	-0.42	0	85	3.3e-009	1	U	K.VVAATTAQIQEFTK.Y
1558	129	–	142	753.9115	1505.8084	1505.8090	-0.37	0	51	8e-006	1	U	K.VVAATTAQIQEFTK.Y
1559	129	–	142	753.9115	1505.8085	1505.8090	-0.31	0	79	1.2e-008	1	U	K.VVAATTAQIQEFTK.Y
1561	129	–	142	753.9116	1505.8086	1505.8090	-0.23	0	38	0.00016	1	U	K.VVAATTAQIQEFTK.Y
1562	129	–	142	753.9117	1505.8088	1505.8090	-0.11	0	37	0.00018	1	U	K.VVAATTAQIQEFTK.Y
1563	129	–	142	753.9117	1505.8088	1505.8090	-0.088	0	85	2.9e-009	1	U	K.VVAATTAQIQEFTK.Y
1564	129	–	142	753.9119	1505.8093	1505.8090	0.22	0	59	1.2e-006	1	U	K.VVAATTAQIQEFTK.Y
1565	129	–	142	502.9437	1505.8093	1505.8090	0.25	0	39	0.00013	1	U	K.VVAATTAQIQEFTK.Y
1566	129	–	142	753.9152	1505.8159	1505.8090	4.63	0	88	1.5e-009	1	U	K.VVAATTAQIQEFTK.Y
835	143	–	153	580.2813	1158.5480	1158.5491	-1.02	0	32	0.00068	1	U	K.YAGIAATAYCR.S
980	143	–	153	608.7927	1215.5708	1215.5706	0.16	0	23	0.0055	1	U	K.YAGIAATAYCR.S + Carbamidomethyl (C)
983	143	–	153	608.7929	1215.5712	1215.5706	0.45	0	22	0.0057	1	U	K.YAGIAATAYCR.S + Carbamidomethyl (C)

Query	Start	–	End	Observed	Mr(expt)	Mr(calc)	ppm	M	Score	Expect	Rank	U	Peptide
984	143	–	153	608.7929	1215.5712	1215.5706	0.49	0	36	0.00025	1	U	K.YAGIAATAYCR.S + Carbamidomethyl (C)
985	143	–	153	608.7929	1215.5712	1215.5706	0.50	0	26	0.0023	1	U	K.YAGIAATAYCR.S + Carbamidomethyl (C)
986	143	–	153	608.7929	1215.5713	1215.5706	0.55	0	80	8.9e-009	1	U	K.YAGIAATAYCR.S + Carbamidomethyl (C)
989	143	–	153	608.7930	1215.5715	1215.5706	0.73	0	30	0.0011	1	U	K.YAGIAATAYCR.S + Carbamidomethyl (C)
993	143	–	153	608.7932	1215.5718	1215.5706	0.96	0	41	8.2e-005	1	U	K.YAGIAATAYCR.S + Carbamidomethyl (C)
748	161	–	168	562.2363	1122.4580	1122.4587	-0.62	0	28	0.0014	1	U	K.WDCVQCQK.W + 2 Carbamidomethyl (C)
749	161	–	168	562.2364	1122.4583	1122.4587	-0.35	0	29	0.0013	1	U	K.WDCVQCQK.W + 2 Carbamidomethyl (C)
750	161	–	168	562.2364	1122.4583	1122.4587	-0.33	0	31	0.00078	1	U	K.WDCVQCQK.W + 2 Carbamidomethyl (C)
8	169	–	174	351.1847	700.3549	700.3544	0.65	0	20	0.011	1	U	K.WVPDGK.I
3050	175	–	192	672.0333	2013.0781	2013.0783	-0.094	0	43	4.8e-005	1	U	K.IITTFTSLLSDTNGYVLR.S
3051	175	–	192	1007.5464	2013.0782	2013.0783	-0.0070	0	66	2.3e-007	1	U	K.IITTFTSLLSDTNGYVLR.S
3052	175	–	192	1007.5469	2013.0792	2013.0783	0.49	0	75	3.5e-008	1	U	K.IITTFTSLLSDTNGYVLR.S
3053	175	–	192	672.0341	2013.0805	2013.0783	1.11	0	45	3.2e-005	1	U	K.IITTFTSLLSDTNGYVLR.S
362	198	–	204	456.2710	910.5274	910.5276	-0.25	0	32	0.00057	1	U	K.TIYLVFR.G
365	198	–	204	456.2711	910.5277	910.5276	0.10	0	35	0.00031	1	U	K.TIYLVFR.G
368	198	–	204	456.2714	910.5282	910.5276	0.59	0	36	0.00024	1	U	K.TIYLVFR.G
2922	211	–	227	972.5085	1943.0025	1943.0040	-0.80	0	73	4.6e-008	1	U	R.SAITDIVFNFSDYKPVK.G
2923	211	–	227	972.5086	1943.0027	1943.0040	-0.69	0	65	3e-007	1	U	R.SAITDIVFNFSDYKPVK.G
2925	211	–	227	648.6763	1943.0070	1943.0040	1.54	0	47	1.9e-005	1	U	R.SAITDIVFNFSDYKPVK.G
2926	211	–	227	648.6771	1943.0094	1943.0040	2.77	0	20	0.01	1	U	R.SAITDIVFNFSDYKPVK.G
2927	211	–	227	648.6773	1943.0100	1943.0040	3.06	0	57	2.1e-006	1	U	R.SAITDIVFNFSDYKPVK.G
5256	231	–	261	1189.5903	3565.7491	3565.7518	-0.77	0	42	6.7e-005	1	U	K.VHAGFLSSYEQVVNDYFPVVQEQLTAHPTYK.V
5257	231	–	261	1189.5904	3565.7494	3565.7518	-0.68	0	63	5.5e-007	1	U	K.VHAGFLSSYEQVVNDYFPVVQEQLTAHPTYK.V
5258	231	–	261	892.4458	3565.7542	3565.7518	0.68	0	45	3.3e-005	1	U	K.VHAGFLSSYEQVVNDYFPVVQEQLTAHPTYK.V
5259	231	–	261	892.4463	3565.7559	3565.7518	1.16	0	31	0.00074	1	U	K.VHAGFLSSYEQVVNDYFPVVQEQLTAHPTYK.V
3844	262	–	284	790.7572	2369.2499	2369.2525	-1.12	0	39	0.00014	1	U	K.VIVTGHSLGGAQALLAGMDLYQR.E
3846	262	–	284	1185.6324	2369.2502	2369.2525	-0.97	0	87	1.9e-009	1	U	K.VIVTGHSLGGAQALLAGMDLYQR.E
3847	262	–	284	1185.6324	2369.2502	2369.2525	-0.97	0	101	8.1e-011	1	U	K.VIVTGHSLGGAQALLAGMDLYQR.E
3852	262	–	284	790.7578	2369.2516	2369.2525	-0.40	0	52	5.8e-006	1	U	K.VIVTGHSLGGAQALLAGMDLYQR.E
3853	262	–	284	790.7578	2369.2516	2369.2525	-0.39	0	51	8.4e-006	1	U	K.VIVTGHSLGGAQALLAGMDLYQR.E
3855	262	–	284	790.7579	2369.2520	2369.2525	-0.23	0	50	1.1e-005	1	U	K.VIVTGHSLGGAQALLAGMDLYQR.E
3857	262	–	284	790.7581	2369.2525	2369.2525	-0.0068	0	19	0.013	1	U	K.VIVTGHSLGGAQALLAGMDLYQR.E
3858	262	–	284	790.7582	2369.2527	2369.2525	0.069	0	49	1.2e-005	1	U	K.VIVTGHSLGGAQALLAGMDLYQR.E
3859	262	–	284	790.7584	2369.2534	2369.2525	0.35	0	51	7.1e-006	1	U	K.VIVTGHSLGGAQALLAGMDLYQR.E
3860	262	–	284	790.7586	2369.2540	2369.2525	0.63	0	23	0.0047	1	U	K.VIVTGHSLGGAQALLAGMDLYQR.E
3861	262	–	284	790.7588	2369.2545	2369.2525	0.84	0	49	1.2e-005	1	U	K.VIVTGHSLGGAQALLAGMDLYQR.E
3862	262	–	284	593.3211	2369.2551	2369.2525	1.09	0	31	0.0008	1	U	K.VIVTGHSLGGAQALLAGMDLYQR.E
3889	262	–	284	597.3186	2385.2451	2385.2475	-0.98	0	25	0.0031	1	U	K.VIVTGHSLGGAQALLAGMDLYQR.E + Oxidation (M)
3890	262	–	284	796.0891	2385.2455	2385.2475	-0.82	0	34	0.00039	1	U	K.VIVTGHSLGGAQALLAGMDLYQR.E + Oxidation (M)
3892	262	–	284	796.0892	2385.2458	2385.2475	-0.71	0	26	0.0025	1	U	K.VIVTGHSLGGAQALLAGMDLYQR.E + Oxidation (M)
3897	262	–	284	796.0893	2385.2461	2385.2475	-0.58	0	45	3.5e-005	1	U	K.VIVTGHSLGGAQALLAGMDLYQR.E + Oxidation (M)
3900	262	–	284	796.0895	2385.2468	2385.2475	-0.30	0	27	0.0021	1	U	K.VIVTGHSLGGAQALLAGMDLYQR.E + Oxidation (M)
3901	262	–	284	796.0896	2385.2471	2385.2475	-0.16	0	23	0.005	1	U	K.VIVTGHSLGGAQALLAGMDLYQR.E + Oxidation (M)
3902	262	–	284	796.0898	2385.2477	2385.2475	0.082	0	46	2.6e-005	1	U	K.VIVTGHSLGGAQALLAGMDLYQR.E + Oxidation (M)
3903	262	–	284	796.0898	2385.2477	2385.2475	0.11	0	52	6.2e-006	1	U	K.VIVTGHSLGGAQALLAGMDLYQR.E + Oxidation (M)
3904	262	–	284	796.0899	2385.2478	2385.2475	0.14	0	62	5.9e-007	1	U	K.VIVTGHSLGGAQALLAGMDLYQR.E + Oxidation (M)
3905	262	–	284	796.0901	2385.2486	2385.2475	0.48	0	57	2e-006	1	U	K.VIVTGHSLGGAQALLAGMDLYQR.E + Oxidation (M)
4667	262	–	287	923.4866	2767.4380	2767.4439	-2.14	1	29	0.0011	1	U	K.VIVTGHSLGGAQALLAGMDLYQREPR.L + Oxidation (M)
4668	262	–	287	923.4873	2767.4402	2767.4439	-1.34	1	36	0.00026	1	U	K.VIVTGHSLGGAQALLAGMDLYQREPR.L + Oxidation (M)

Query	Start	–	End	Observed	Mr(expt)	Mr(calc)	ppm	M	Score	Expect	Rank	U	Peptide
4669	262	–	287	692.8674	2767.4405	2767.4439	-1.25	1	48	1.7e-005	1	U	K.VIVTGHSLGGAQALLAGMDLYQREPR.L + Oxidation (M)
4670	262	–	287	554.4955	2767.4412	2767.4439	-1.00	1	20	0.011	1	U	K.VIVTGHSLGGAQALLAGMDLYQREPR.L + Oxidation (M)
4671	262	–	287	692.8677	2767.4419	2767.4439	-0.75	1	38	0.00017	1	U	K.VIVTGHSLGGAQALLAGMDLYQREPR.L + Oxidation (M)
1799	288	–	302	529.3062	1584.8969	1584.8988	-1.19	1	30	0.00092	1	U	R.LSPKNLSIFTVGGPR.V
1800	288	–	302	529.3065	1584.8977	1584.8988	-0.68	1	31	0.00086	1	U	R.LSPKNLSIFTVGGPR.V
839	292	–	302	580.8245	1159.6345	1159.6350	-0.41	0	21	0.008	1	U	K.NLSIFTVGGPR.V
840	292	–	302	580.8245	1159.6345	1159.6350	-0.41	0	19	0.012	1	U	K.NLSIFTVGGPR.V
841	292	–	302	580.8246	1159.6346	1159.6350	-0.31	0	59	1.1e-006	1	U	K.NLSIFTVGGPR.V
842	292	–	302	580.8246	1159.6347	1159.6350	-0.24	0	21	0.0076	1	U	K.NLSIFTVGGPR.V
845	292	–	302	580.8248	1159.6351	1159.6350	0.16	0	56	2.4e-006	1	U	K.NLSIFTVGGPR.V
847	292	–	302	580.8257	1159.6368	1159.6350	1.59	0	57	1.8e-006	1	U	K.NLSIFTVGGPR.V
848	292	–	302	580.8265	1159.6384	1159.6350	2.94	0	52	7e-006	1	U	K.NLSIFTVGGPR.V
3356	303	–	321	716.0237	2145.0492	2145.0531	-1.83	0	34	0.00036	1	U	R.VGNPTFAYYVESTGIPFQR.T
3357	303	–	321	1073.5321	2145.0496	2145.0531	-1.62	0	61	7.8e-007	1	U	R.VGNPTFAYYVESTGIPFQR.T
3362	303	–	321	1073.5322	2145.0498	2145.0531	-1.52	0	31	0.00071	1	U	R.VGNPTFAYYVESTGIPFQR.T
3369	303	–	321	1073.5328	2145.0510	2145.0531	-0.96	0	21	0.0081	1	U	R.VGNPTFAYYVESTGIPFQR.T
3379	303	–	321	1073.5334	2145.0522	2145.0531	-0.40	0	19	0.014	1	U	R.VGNPTFAYYVESTGIPFQR.T
3386	303	–	321	716.0253	2145.0539	2145.0531	0.38	0	21	0.0083	1	U	R.VGNPTFAYYVESTGIPFQR.T
3388	303	–	321	1073.5345	2145.0544	2145.0531	0.62	0	27	0.002	1	U	R.VGNPTFAYYVESTGIPFQR.T
3389	303	–	321	1073.5346	2145.0546	2145.0531	0.71	0	72	5.7e-008	1	U	R.VGNPTFAYYVESTGIPFQR.T
3390	303	–	321	1073.5346	2145.0546	2145.0531	0.71	0	60	9.9e-007	1	U	R.VGNPTFAYYVESTGIPFQR.T
3391	303	–	321	1073.5351	2145.0556	2145.0531	1.18	0	28	0.0016	1	U	R.VGNPTFAYYVESTGIPFQR.T
3392	303	–	321	716.0265	2145.0576	2145.0531	2.08	0	34	0.00036	1	U	R.VGNPTFAYYVESTGIPFQR.T
4634	326	–	349	686.3691	2741.4475	2741.4442	1.18	1	19	0.013	1	U	K.RDIVPHVPPQSFGFLHPGVESWIK.S
4259	327	–	349	862.7878	2585.3417	2585.3431	-0.56	0	29	0.0013	1	U	R.DIVPHVPPQSFGFLHPGVESWIK.S
4284	327	–	349	647.3456	2585.3533	2585.3431	3.92	0	22	0.007	1	U	R.DIVPHVPPQSFGFLHPGVESWIK.S
4285	327	–	349	647.3458	2585.3543	2585.3431	4.31	0	27	0.002	1	U	R.DIVPHVPPQSFGFLHPGVESWIK.S
2195	350	–	365	877.4162	1752.8179	1752.8200	-1.17	0	59	1.2e-006	1	U	K.SGTSNVQICTSEIETK.D + Carbamidomethyl (C)
2217	350	–	365	585.2812	1752.8217	1752.8200	0.96	0	22	0.0062	1	U	K.SGTSNVQICTSEIETK.D + Carbamidomethyl (C)
